# Co-exposure to *Anaplasma* spp., *Coxiella burnetii* and tick-borne encephalitis virus in sheep in southern Germany

**DOI:** 10.1186/s13028-022-00659-6

**Published:** 2023-02-15

**Authors:** Benjamin Ulrich Bauer, Martin Runge, Melanie Schneider, Laura Könenkamp, Imke Steffen, Wiebke Rubel, Martin Ganter, Clara Schoneberg

**Affiliations:** 1 Clinic for Swine and Small Ruminants, Forensic Medicine and Ambulatory Service, University of Veterinary Medicine Hannover, Foundation, Bischofsholer Damm 15, 30173 Hannover, Germany; 2 Food and Veterinary Institute Braunschweig/Hannover, Lower Saxony State Office for Consumer Protection and Food Safety (LAVES), Eintrachtweg 17, 30173 Hannover, Germany; 3 Institute for Biochemistry and Research Center for Emerging Infections and Zoonoses, University of Veterinary Medicine Hannover, Foundation, Bünteweg 17, 30559 Hannover, Germany; 4 Department of Biometry, Epidemiology and Information Processing, WHO Collaborating Centre for Research and Training Health at the Human-Animal-Environment Interface, University of Veterinary Medicine Hannover, Foundation, Bünteweg 2, 30559 Hannover, Germany

**Keywords:** *Anaplasma phagocytophilum*, *Anaplasma ovis*, *Dermacentor marginatus*, *Flaviviridae*, *Ixodes ricinus*, Tick-borne encephalitis, Tick-borne fever, Q fever, Zoonosis

## Abstract

The intracellular bacteria *Anaplasma* spp. and *Coxiella burnetii* and the tick-borne encephalitis virus (TBEV) are tick-transmitted pathogens circulating in the southern German sheep population. Knowledge of interaction among *Anaplasma* spp., *C. burnetii* and TBEV in sheep is lacking, but together they might promote and reinforce disease progression. The current study aimed to identify co-exposure of sheep to *Anaplasma* spp., *C. burnetii* and TBEV. For this purpose, 1,406 serum samples from 36 sheep flocks located in both southern German federal states, Baden-Wuerttemberg and Bavaria, were analysed by ELISAs to determine the antibody levels of the three pathogens. Inconclusive and positive results from the TBEV ELISA were additionally confirmed by a serum neutralisation assay. The proportion of sheep with antibodies against *Anaplasma* spp. (47.2%), *C. burnetii* (3.7%) and TBEV (4.7%) differed significantly. Significantly more flocks with *Anaplasma* spp. seropositive sheep (91.7%) were detected than flocks with antibodies against TBEV (58.3%) and *C. burnetii* (41.7%), but there was no significant difference between the number of flocks which contained TBEV and *C. burnetii* seropositive sheep. Seropositivity against at least two pathogens was detected in 4.7% of sheep from 20 flocks. Most co-exposed sheep had antibodies against *Anaplasma* spp./TBEV (n = 36), followed by *Anaplasma* spp./*C. burnetii* (n = 27) and *Anaplasma* spp./*C. burnetii*/TBEV (n = 2). Only one sheep showed an immune response against *C. burnetii* and TBEV. Flocks with sheep being positive against more than one pathogen were widely distributed throughout southern Germany. The descriptive analysis revealed no association between the antibody response of the three pathogens at animal level. Taking the flocks as a cluster variable into account, the exposure to TBEV reduced the probability of identifying *C. burnetii* antibodies in sheep significantly (odds ratio 0.46; 95% confidence interval 0.24–0.85), but the reason for this is unknown. The presence of *Anaplasma* spp. antibodies did not influence the detection of antibodies against *C. burnetii* and TBEV. Studies under controlled conditions are necessary to evaluate any possible adverse impact of co-exposure to tick-borne pathogens on sheep health. This can help to clarify rare disease patterns. Research in this field may also support the One Health approach due to the zoonotic potential of *Anaplasma* spp., *C. burnetii* and TBEV.

## Findings

The intracellular bacteria *Coxiella burnetii, Anaplasma phagocytophilum* and *Anaplasma ovis*, and the tick-borne encephalitis virus (TBEV, *Flaviviridae*) are tick-transmitted pathogens and circulate in sheep flocks in the southern German federal states, Baden-Wuerttemberg (BW) and Bavaria (BAV) [1–3]. These pathogens also have a zoonotic potential and can cause illness in humans, such as flu-like symptoms and neurological disorders [4–7]. The main vector of *A. phagocytophilum* and TBEV is *Ixodes ricinus* and this tick species is widely distributed throughout Germany [4, 8, 9]. *C. burnetii* has also been found in *I. ricinus* [10], but *Dermacentor marginatus* is considered to transmit this pathogen to sheep [11, 12]. The existence of *D. marginatus* is limited to certain areas in southern Germany [8]. Recently, *A. ovis* was identified in engorged *D. marginatus* from Bavarian sheep, but this does not prove its vector competence, and solid data about *A. ovis* vectors are still lacking [3]. The clinical signs of these tick-borne pathogens are diverse in sheep. An infection with *C. burnetii* can result in reproductive disorders [5]. Haemolytic anaemia is caused by *A. ovis*, whereas an *A. phagocytophilum* infection results in tick-borne fever [13]. Moreover, *A. phagocytophilum* is an immunosuppressive agent and negatively affects the function of neutrophils, resulting in a higher susceptibility to secondary infections [14]. TBEV infection seems to be asymptomatic, but neurological signs in sheep have been reported [2, 15]. Concurrent infections of the louping ill virus (LIV, *Flaviviridae*) with *A. phagocytophilum* promote the onset of severe LIV-associated neurological disorders [16]. Furthermore, a dual infection of *A. phagocytophilum* and TBEV resulted in a significantly higher TBEV antibody response compared to a consecutive infection [15]. However, knowledge of interaction among *Anaplasma* spp., *C. burnetii* and TBEV in sheep is lacking, but together they might promote and reinforce disease progression.

**Fig. 1:**
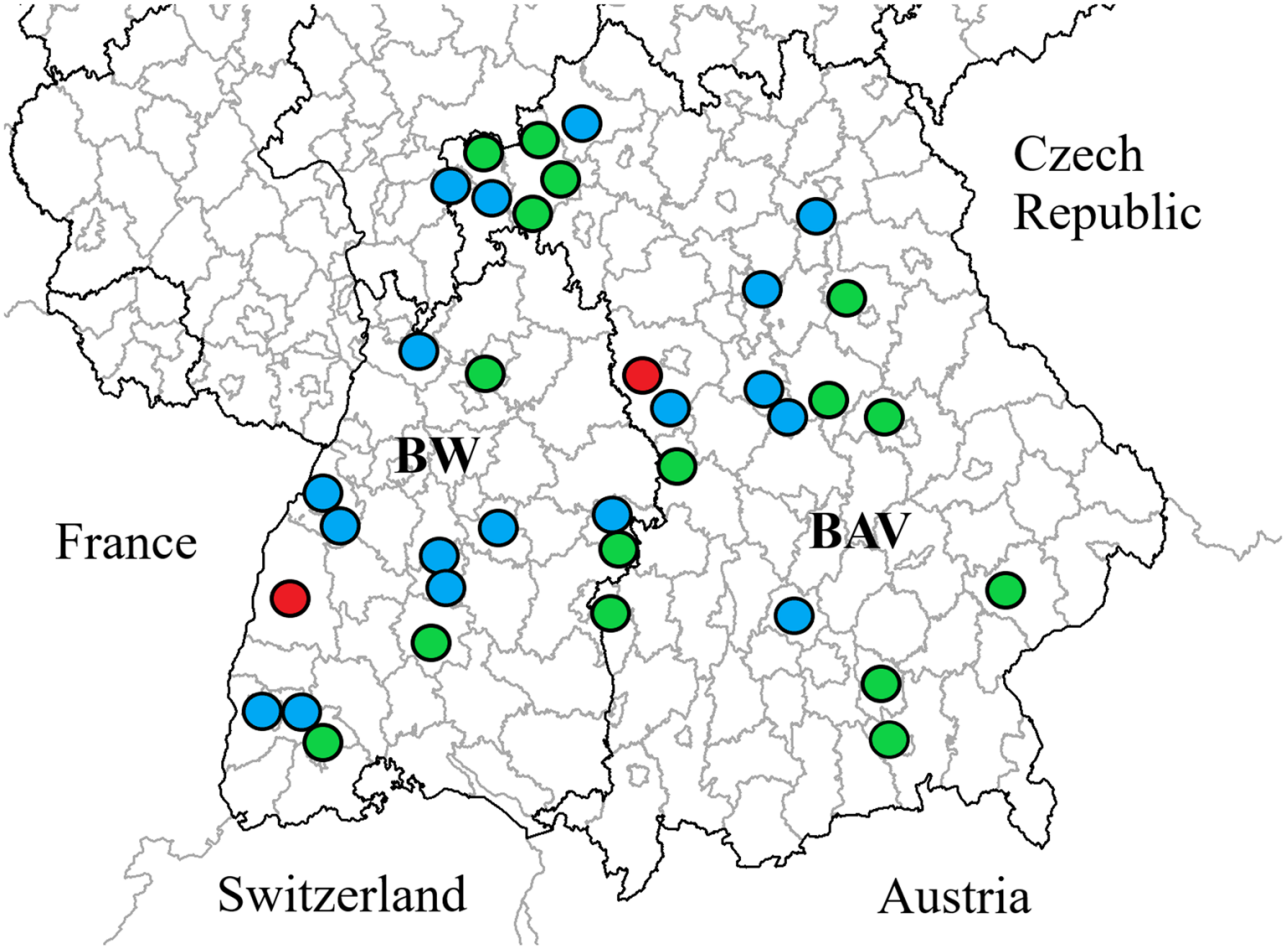
Location of 36 examined sheep flocks in southern Germany. Concurrent positive antibody levels against *Anaplasma* spp., *Coxiella burnetii* and tick-borne encephalitis virus were determined in individual sheep in two flocks (red). Co-exposure to two pathogens at animal level were identified in 18 sheep flocks (blue), whereas no co-exposed sheep were detected in 16 flocks (green). BW: Baden-Wuerttemberg, BAV: Bavaria

The current study aimed to identify to which extent grazing sheep had antibodies against *Anaplasma* spp., *C. burnetii* and TBEV. For this purpose, 1,406 serum samples from 36 sheep flocks located in BW and BAV were analysed to detect antibodies against the three tick-borne pathogens. Initially, the blood samples were collected for a Q fever study, and the number of specimens required from each flock to estimate the positivity rate was calculated on the assumption of 3% expected prevalence, 95% confidence interval, 80% power and 5% precision [1]. A maximum of 44 animals per flock were sampled between November 2017 and June 2018. The blood sampling was performed in accordance with high ethical standards and approved by the federal state governments. The locations of the flocks are presented in Fig. 1.

Antibodies against *Anaplasma* spp., *C. burnetii* and TBEV were determined by three different commercial ELISAs in accordance with the manufacturers’ instructions and described in detail elsewhere [1, 17, 18]. An inhibition of ≥ 30% was assessed as positive for the *Anaplasma* spp. assay (Anaplasma Antibody Test Kit, cELISA v2, VMRD, Inc., Pullman, WA, USA), but this ELISA does not differentiate between antibodies against *A. phagocytophilum* and *A. ovis*. A sensitivity of 91.9% and a specificity of 86.9% were assumed according to Shabana et al. [19]. A S/P (%) > 40 for the *C. burnetii* ELISA was considered positive (Q Fever Antibody Test Kit, IDEXX Switzerland AG, Liebefeld, Switzerland), in accordance with the sensitivity and specificity of 100% each stated by the manufacturer. Regarding the TBEV ELISA (Immunozym^®^ FSME IgG all Species, PROGEN Biotechnik GmbH, Heidelberg, Germany), the manufacturer specified samples with > 126 Vienna Units (VIEU)/mL as positive; values between 63 and 126 VIEU/mL were classified as inconclusive. A sensitivity of 97% and a specificity of 99% were assumed in accordance with the product information. The inconclusive and positive samples were confirmed with a serum neutralisation assay as recently described, and antibody titres of ≥ 1:40 were counted as positive [17].

The test results and their agreement were evaluated in descriptive tables. To determine the true prevalence at animal and flock level, the apparent prevalence was corrected for misclassification probabilities (sensitivity and specificity of the diagnostic tests) using the Rogan-Gladen estimator [20]. The prevalence of antibodies against more than one pathogen in the same individual or flock was also adjusted by correcting the test accuracies for parallel testing [21]. In addition, the proportion of positive antibody results of the three pathogens at animal and flock level was compared by Fisher’s exact test. Subsequently, a logistic regression that considered the antibody result of one pathogen as the outcome and the other pathogen as the risk factor as well as the flocks as a cluster variable, was performed for the binary test results at animal level. The results of the two antibody tests were analysed in a logistic regression model to detect a significant association between pathogen exposure. Odds ratios were calculated to determine the strength and direction of a possible association. The association of the test results at flock level was analysed using Fisher’s exact test. A P-value of < 0.05 was considered significant. For all calculations, the statistical software SAS (SAS Institute Inc., Cary, NC, USA) was used.

There was a significant difference among the results of true seroprevalence between *Anaplasma* spp., *C. burnetii* and TBEV at animal level (*p* < 0.05). Most sheep had antibodies against *Anaplasma* spp. (47.2%), followed by TBEV (4.7%) and *C. burnetii* (3.7%). Significantly more flocks with *Anaplasma* spp. seropositive sheep were detected (n = 33; 91.7%) compared to flocks being seropositive for TBEV (n = 21; 58.3%) and *C. burnetii* (n = 15; 41.7%) (*p* < 0.05), but there was no significant different between flocks which contained TBEV and *C. burnetii* seropositive sheep. Seropositivity against at least two pathogens was detected in 66 (4.7%) sheep from 20 flocks (55.6%). The flocks with co-exposed sheep were widely distributed throughout southern Germany. Details of sheep and flocks with antibodies against more than one of the pathogens are presented in Table 1; Fig. 1, respectively. The descriptive analysis revealed no association of the three pathogens at animal level (Table 2). Taking the flocks as a cluster variable into account, there was a significant association between presence of *C. burnetii* and TBEV antibodies. The exposure to TBEV reduced the probability of identifying *C. burnetii* antibodies in sheep by half (Table 3). The presence of *Anaplasma* spp. antibodies did not influence the antibody detection of *C. burnetii* and TBEV. Based on current knowledge, *A. ovis* appeared only locally in one sheep flock from northern Bavaria, but this flock did not participate in the current study [3]. Therefore, most *Anaplasma* spp. antibodies were possibly induced by *A. phagocythophilum* due to the wide dissemination in the German sheep population [3, 22, 23]. This was taken into account while interpreting the presented findings.

**Table 1:** Seropositivity of sheep against at least two tick-borne pathogens determined by serological assays

Co-exposure of tick-borne pathogens	Number of antibody positive sheep
*Anaplasma* spp./*C. burnetii*	27
*C. burnetii*/TBEV	1
*Anaplasma* spp./TBEV	36
*Anaplasma* spp.*/C. burnetii/*TBEV	2

In total, 66 sheep (n = 1,406) in 20 flocks (n = 36) from southern Germany had antibodies against at least two tick-borne pathogens. The corrected seroprevalence at animal level was considered. *C. burnetii: Coxiella burnetii*, TBEV: tick-borne encephalitis virus

**Table 2:** Association between antibodies against three tick-borne pathogens in sheep at animal level

Pathogen 1	Pathogen 2	Cohen’s Kappa	*p*-value
*C. burnetii*	*Anaplasma* spp.	0.0081	0.48
*C. burnetii*	TBEV	0.0025	0.76
*Anaplasma* spp.	TBEV	0.0036	0.81

Results were evaluated by descriptive analysis and the *p*-values referred to the Fisher’s exact test. (*p* < 0.05). *C. burnetii*: *Coxiella burnetii*, TBEV: tick-borne encephalitis virus

**Table 3:** Association between antibodies against three tick-borne pathogens in sheep at animal level with flock as a cluster variable

Dependent variable	Independent variable	Odds ratio [95% confidence interval]	*p*-value
*C. burnetii*	*Anaplasma* spp.	0.93 [0.53–1.64]	0.81
*C. burnetii*	TBEV	0.46 [0.24–0.85]	0.01
*Anaplasma* spp.	TBEV	1.25 [0.90–1.74]	0.17

Results were evaluated by logistic regression analysis with flock as a cluster variable, and the *p*-values referred to the corresponding Chi square test (*p* < 0.05). *C. burnetii*: *Coxiella burnetii*, TBEV: tick-borne encephalitis virus

The significant differences among antibody rates of *Anaplasma* spp., TBEV and *C. burnetii* at sheep level might correlate with the presence of the pathogens in ticks collected in southern Germany. Up to 8.3% of questing *I. ricinus* contained *A. phagocytophilum* [24], whereas the detection rate of TBEV ranged from 0 to 5.3% [25], and *C. burnetii* has been determined only in one engorged *D. marginatus* so far [12, 26]. Information about natural co-exposure with *Anaplasma* spp., *C. burnetii* and TBEV in sheep is extremely rare [27]. Significantly fewer sheep with *C. burnetii* antibodies were detected in flocks which also had sheep with antibodies against TBEV. Knowledge of interaction between both pathogens is missing, and we can only speculate about the possible reciprocal influence of both pathogens in sheep flocks. This observation needs further investigation in the future. The humoral immune response against *Anaplasma* spp., *C. burnetii* and TBEV lasts for several months in sheep [17, 28, 29]. Furthermore, co-infection with various pathogens were described for different tick species, but findings on the coincidental infection with *Anaplasma* spp., *C. burnetii* or TBEV are seldom in ticks [30–32]. Considering both these circumstances, we assume that natural exposure of sheep occurs through infestation of different ticks infected with one of the above-mentioned agents. In addition, infections with the pathogens might occur consecutively rather than at the same time. This is supported by previous findings of Paulsen et al. [15], who demonstrated that only a simultaneous infection of *A. phagocytophilum* and TBEV in lambs resulted in a significantly higher TBEV antibody response, but a consecutive infection had no influence on the antibody response of both pathogens. In the current study, the presence of *Anaplasma* spp. antibodies also did not influence the TBEV antibody detection at animal and flock level. Nevertheless, the number of lambs suffering from tick-borne encephalitis (TBE) seems to be on the increase in southern Germany [33], and the immunosuppressive impact of *A. phagocytophilum* should be investigated in lambs naturally infected with TBEV. Despite the fact that a detrimental influence was not confirmed after experimental infection with TBEV, the artificially infected lambs did not develop clinical signs of TBE [15]. Therefore, the outbreak of TBE in sheep appears to depend on as yet unknown factors.

The authors are aware of the limitations of the present study. The antibody response against the three tick-borne pathogens is the result of a natural exposure in the field in the past. Therefore, it is impossible to determine the exact time of infection and the possibly related clinical impact. Moreover, we cannot rule out that false-seropositive sheep were included in the evaluation because of an estimated specificity of 86.9% [19] of the *Anaplasma* spp. ELISA, and a possibly reduced specificity of the *C. burnetii* ELISA [34]. Only the inconclusive and positive results from the TBEV ELISA were confirmed by a serum neutralisation assay, which is considered as gold standard for TBEV antibody detection [17]. This minimises the risk of sheep being tested false-seropositive for TBEV antibodies. All in all, the low numbers of co-exposures could also be the consequence of imperfect specificity of the diagnostic tests used. In the future, co-infection has to be determined by further tests such as molecular assays to detect pathogen DNA. Nevertheless, our findings contribute to the complex issue of tick-borne pathogens in sheep. Co-exposure to *Anaplasma* spp., *C. burnetii* and TBEV seems to be sporadic among grazing sheep flocks in southern Germany. The further spread of TBEV and the emerging onset of *A. ovis* in Germany might increase cases of co-infection [3, 4]. More targeted investigations are needed to evaluate an adverse impact of co-exposure to tick-borne pathogens on sheep health due to the fact that *A. phagocytophilum* influences the immune response and disease progression of concurrent flavivirus infections [15, 16]. In addition, sheep flocks can be implemented as sentinels to identify potential new risk areas of emerging zoonotic pathogens such as TBEV [17]. Therefore, further research in this field may also support the One Health approach.

## Data Availability

The datasets used and/or analysed during the current study are available from the corresponding author on reasonable request.

## Abbreviations

BAV: Bavaria
BW: Baden-Wuerttemberg
cELISA: Competitive enzyme-linked immunosorbent assay
ELISA: Enzyme-linked immunosorbent assay
LIV: Louping ill virus
S/P (%): Sample/positive percentage
TBE: Tick-borne encephalitis
TBEV: Tick-borne encephalitis virus
VIEU: Vienna units

## References

[1] Wolf A, Prüfer TL, Schoneberg C, Campe A, Runge M, Ganter M, Bauer BU (2020). Prevalence of *Coxiella burnetii* in German sheep flocks and evaluation of a novel approach to detect an infection via preputial swabs at herd-level. Epidemiol Infect.

[2] Böhm B, Schade B, Bauer B, Hoffmann B, Hoffmann D, Ziegler U, Beer M, Klaus C, Weissenböck H, Böttcher J (2017). Tick-borne encephalitis in a naturally infected sheep. BMC Vet Res.

[3] Bauer BU, Răileanu C, Tauchmann O, Fischer S, Ambros C, Silaghi C, Ganter M (2021). *Anaplasma**phagocytophilum*and*Anaplasma**ovis*—emerging pathogens in the German sheep population. Pathogens (Basel, Switzerland).

[4] Hellenbrand W, Kreusch T, Böhmer MM, Wagner-Wiening C, Dobler G, Wichmann O, Altmann D (2019). Epidemiology of tick-borne encephalitis (TBE) in Germany, 2001–2018. Pathogens (Basel, Switzerland).

[5] Bauer BU, Runge M, Campe A, Henning K, Mertens-Scholz K, Boden K, Sobotta K, Frangoulidis D, Knittler MR, Matthiesen S (2020). *Coxiella burnetii*: a review focusing on infections in German sheep and goat flocks. Berl Munch Tierarztl Wochenschr.

[6] Matei IA, Estrada-Peña A, Cutler SJ, Vayssier-Taussat M, Varela-Castro L, Potkonjak A, Zeller H, Mihalca AD (2019). A review on the eco-epidemiology and clinical management of human granulocytic anaplasmosis and its agent in Europe. Parasit Vectors.

[7] Chochlakis D, Ioannou I, Tselentis Y, Psaroulaki A (2010). Human anaplasmosis and *Anaplasma ovis* variant. Emerg Infect Dis.

[8] Rubel F, Brugger K, Chitimia-Dobler L, Dautel H, Meyer-Kayser E, Kahl O (2021). Atlas of ticks (Acari: Argasidae, Ixodidae) in Germany. Exp Appl Acarol.

[9] Stuen S, Granquist E, Silaghi C (2013). *Anaplasma**phagocytophilum*—a widespread multi-host pathogen with highly adaptive strategies. Front Cell Infect Microbiol.

[10] Hildebrandt A, Straube E, Neubauer H, Schmoock G (2011). *Coxiella burnetii* and coinfections in *Ixodes ricinus* ticks in central Germany. Vector Borne Zoonotic Dis.

[11] Körner S, Makert GR, Ulbert S, Pfeffer M, Mertens-Scholz K (2021). The prevalence of* Coxiella burnetii* in hard ticks in Europe and their role in Q Fever transmission revisited—a systematic review. Front Vet Sci.

[12] Sting R, Breitling N, Oehme R, Kimmig P (2004). Untersuchungen zum Vorkommen von *Coxiella burnetii* bei Schafen und Zecken der Gattung Dermacentor in Baden-Wuerttemberg [*Studies on the prevalence of Coxiella burnetii in sheep and ticks of the genus Dermacentor in Baden-Wuerttemberg*]. Deut Tieraertzl Woch.

[13] Stuen S (2016). Haemoparasites in small ruminants in European countries: challenges and clinical relevance. Small Rumin Res.

[14] Woldehiwet Z (2006). *Anaplasma phagocytophilum* in ruminants in Europe. Ann N Y Acad Sci.

[15] Paulsen KM, Granquist EG, Okstad W, Vikse R, Stiasny K, Andreassen AK, Stuen S (2019). Experimental infection of lambs with tick-borne encephalitis virus and co-infection with *Anaplasma phagocytophilum*. PLoS ONE.

[16] Reid HW, Buxton D, Pow I, Brodie TA, Holmes PH, Urquhart GM (1986). Response of sheep to experimental concurrent infection with tick-borne fever (*Cytoecetes phagocytophila*) and louping-ill virus. Res Vet Sci.

[17] Bauer BU, Könenkamp L, Stöter M, Wolf A, Ganter M, Steffen I, Runge M (2021). Increasing awareness for tick-borne encephalitis virus using small ruminants as suitable sentinels: preliminary observations. One Health.

[18] Rubel W, Schoneberg C, Wolf A, Ganter M, Bauer BU (2021). Seroprevalence and risk factors of *Anaplasma* spp. German small ruminant flocks. Animals.

[19] Shabana II, Alhadlag NM, Zaraket H (2018). Diagnostic tools of caprine and ovine anaplasmosis: a direct comparative study. BMC Vet Res.

[20] Rogan WJ, Gladen B (1978). Estimating prevalence from the results of a screening test. Am J Epidemiol.

[21] Dohoo I, Martin W, Stryhn H (2009). Veterinary epidemiologic research.

[22] Scharf W, Schauer S, Freyburger F, Petrovec M, Schaarschmidt-Kiener D, Liebisch G, Runge M, Ganter M, Kehl A, Dumler JS (2011). Distinct host species correlate with *Anaplasma phagocytophilum* ankA gene clusters. J Clin Microbiol.

[23] Rubel W, Ganter M, Bauer BU (2022). Detection of*Anaplasma phagocytophilum*in ovine serum samples—a retrospective study. Ruminants.

[24] Overzier E, Pfister K, Thiel C, Herb I, Mahling M, Silaghi C (2013). *Anaplasma phagocytophilum* in questing *Ixodes ricinus* ticks: comparison of prevalences and partial 16S rRNA gene variants in urban, pasture, and natural habitats. Appl Environ Microbiol.

[25] Süss J, Schrader C, Falk U, Wohanka N (2004). Tick-borne encephalitis (TBE) in Germany — Epidemiological data, development of risk areas and virus prevalence in field-collected ticks and in ticks removed from humans. Int J Med Microbiol.

[26] Pluta S, Hartelt K, Oehme R, Mackenstedt U, Kimmig P (2010). Prevalence of *Coxiella **burnetii* and *Rickettsia* spp. in ticks and rodents in southern Germany. Ticks Tick-Borne Dis.

[27] Zeman P, Januska J, Orolinova M, Stuen S, Struhar V, Jebavy L (2004). High seroprevalence of granulocytic ehrlichiosis distinguishes sheep that were the source of an alimentary epidemic of tick-borne encephalitis. Wien Klin Wochenschr.

[28] Stuen S, Grøva L, Granquist EG, Sandstedt K, Olesen I, Steinshamn H (2011). A comparative study of clinical manifestations, haematological and serological responses after experimental infection with *Anaplasma phagocytophilum* in two Norwegian sheep breeds. Acta Vet Scand.

[29] Joulié A, Rousset E, Gasqui P, Lepetitcolin E, Leblond A, Sidi-Boumedine K, Jourdain E (2017). *Coxiella burnetii* circulation in a naturally infected flock of sheep: individual follow-up of antibodies in serum and milk. Appl Environ Microbiol.

[30] Pilloux L, Baumgartner A, Jaton K, Lienhard R, Ackermann-Gäumann R, Beuret C, Greub G (2019). Prevalence of *Anaplasma phagocytophilum* and *Coxiella burnetii* in *Ixodes ricinus* ticks in Switzerland: an underestimated epidemiologic risk. New Microbes New Infect.

[31] Ben I, Lozynskyi I (2019). Prevalence of *Anaplasma phagocytophilum* in *Ixodes ricinus* and *Dermacentor reticulatus* and coinfection with *Borrelia burgdorferi* and tick-borne encephalitis virus in western Ukraine. Vector Borne Zoonotic Dis.

[32] Bonnet S, de la Fuente J, Nicollet P, Liu X, Madani N, Blanchard B, Maingourd C, Alongi A, Torina A, Fernández de Mera IG (2013). Prevalence of tick-borne pathogens in adult *Dermacentor* spp. ticks from nine collection sites in France. Vector Borne Zoonotic Dis.

[33] Weber KB, Fast C, Eiden M, Holicki CM, König P, Skuballa J, Köhler K, Bühler M, Kupča A, Ziegler U. Tod auf der Weide - fatale zentralnervöse Erkankung in einer Schafherde [*Death on the pasture—fatal central nervous disease in a sheep flock*]. In: *65 Jahrestagung der DVG-Fachgruppe Pathologie: 04.-06.03.2022 2022; digital*: Deutsche Veterinärmedizinische Gesellschaft e. V.; 2022: 1.

[34] Lurier T, Rousset E, Gasqui P, Sala C, Claustre C, Abrial D, Dufour P, de Crémoux R, Gache K, Delignette-Muller ML (2021). Evaluation using latent class models of the diagnostic performances of three ELISA tests commercialized for the serological diagnosis of *Coxiella burnetii* infection in domestic ruminants. Vet Res.

